# Lymph Node Log-Odds Ratio Accurately Defines Prognosis in Resectable Non-Small Cell Lung Cancer

**DOI:** 10.3390/cancers15072082

**Published:** 2023-03-31

**Authors:** Michal Benej, Thomas Klikovits, Tibor Krajc, Tomas Bohanes, Lisa Schulte, Maximilian Johannes Hochmair, Stefan Watzka, Berta Mosleh, Konrad Hoetzenecker, Clemens Aigner, Mir Alireza Hoda, Michael Rolf Mueller

**Affiliations:** 1 Department of Thoracic Surgery and Karl-Landsteiner-Institute, Clinic Floridsdorf, 1210 Vienna, Austria; 2 Department of Respiratory and Critical Care Medicine, Karl-Landsteiner-Institute for Lung Research and Pulmonary Oncology, Clinic Floridsdorf, 1210 Vienna, Austria; 3 Department of Thoracic Surgery, Medical University of Vienna, 1090 Vienna, Austria

**Keywords:** non-small cell lung cancer, prognosis, lymph node staging, log-odds ratio, lobectomy, surgery

## Abstract

**Simple Summary:**

Non-small cell lung cancer is one of the most common malignancies with major challenges in diagnosis and treatment. Estimating individual prognosis is important in order to tailor the selected therapy to each patient. Lymph node involvement represents a crucial factor which is associated with long-term survival. The currently used classifications of lymph node involvement are anatomically conditioned and do not consider the number of positive lymph nodes and their association with non-involved lymph nodes. In this study we investigated the effect of a mathematical formula (log-odds ratio), including the ratio of positive and negative lymph nodes on survival. We found that this new score is significantly associated with survival in resectable non-small cell lung cancer and might better reflect the individual prognosis.

**Abstract:**

Objectives: The ratio of positive and resected lymph nodes (LN ratio) has been shown to be prognostic in non-small cell lung cancer (NSCLC). Contrary to the LN ratio, calculating the LN log-odds ratio (LN-LOR) additionally considers the total number of resected lymph nodes. We aim to evaluate LN-LOR between positive and resected lymph nodes as a prognostic factor in operable NSCLC. Methods: Patients with NSCLC who underwent curative intent lobectomy treated at two high-volume centers were retrospectively studied. LN-LOR was dichotomized according to impact on OS and further combined with N descriptors and correlated with clinical variables and survival. Results: 944 patients were included. Cut-off analysis revealed that an LN-LOR of −0.34 significantly discriminated patients according to OS (*p* < 0.001, chi-squared test 41.26). When combined with N1 and N2 descriptors, LN-LOR low risk (median OS not reached and 83 months) and LN-LOR high-risk patients (median OS 50 and 59 months) had similar survival irrespective of the anatomical location of the positive lymph nodes. Multivariable Cox regression analysis revealed that age (HR 1.02, 95% CI 1.001–1.032), sex (male, HR 1.65, 95% CI 1.25–2.19), histological subtype (HR 2.11, 95% CI 1.35–3.29), pathological stage (HR 1.23, 95% CI 1.01–1.45) and LN-LOR risk groups (low risk, HR 0.48, 95% CI 0.32–0.72) were independent prognostic factors for OS. Conclusions: This retrospective two-center analysis shows that LN-LOR is significantly associated with OS in resectable NSCLC and might better reflect the biological behavior of the disease, regardless of anatomical lymph node locations. This finding may additionally support the value of extensive LN dissection.

## 1. Introduction

Lymph node (LN) involvement is a well established and strong prognostic factor in resectable non-small cell lung cancer (NSCLC) and represents an integral part of the current TNM staging system [1,2]. The most recent N staging system considers well described anatomical LN locations in order to define clinical and pathological N descriptors [3]. It has been recently demonstrated that patients with multiple affected N1 LN stations have significantly worse overall survival (OS) compared to those with single station N1 disease, and that multiple N2 station involvement is associated with worse OS than single station N2 disease [1]. Importantly, it was also shown that patients with single station N2 and multiple station N1 disease might have similar OS [1]. Thus, considering anatomical LN locations only does not fully reflect disease burden and individual patient prognosis. The number of affected LN stations was consequently proposed to be included in future N descriptor definitions. However, the current recommendations for pathological LN staging in NSCLC do not take the extent of lymphadenectomy or the total number of resected or positive LNs into account. Several previous publications have demonstrated higher numbers of positive LNs or a higher ratio of positive/resected LNs (LN ratio) to be associated with worse prognosis in NSCLC and different other solid tumor types [4,5,6,7,8,9,10]. However, the value of calculating LN ratio (LNR) is limited by the extent of lymphadenectomy and the number of totally resected LNs and there is no value in pN0 patients. The log-odds ratio (LN-LOR) of positive LNs, which mathematically also considers the total number of resected LNs, has been shown to be a strong prognostic factor in several tumor entities [11,12,13,14]. Thus, LN-LOR can serve as a mathematical tool providing a deeper understanding of lymph node involvement in NSCLC after resection and its extrapolation to individual prognosis. We therefore aimed to study the value of LN-LOR as a prognostic factor in resectable NSCLC among different pN subgroups.

## 2. Patients and Methods

### 2.1. Ethics Statement

Retrospective data collection was approved based on the ethical standards prescribed by the Helsinki Declaration of the World Medical Association and with the permission of the Scientific and Research Committee of the City of Vienna (EK22-096-VK) and the Medical University of Vienna (EK1662/2016), which waived the need for individual informed consent for the retrospective data collection.

### 2.2. Study Population

This is a retrospective non-interventional cohort study reviewing prospective databases, including all consecutive patients with complete data who underwent curative intent lobectomy with lymphadenectomy for stage I-III NSCLC with follow-up at two specialized institutions in Vienna, Austria, between 2008 and 2017. Only 24 patients with missing relevant information during the study period have been excluded. In order to investigate a homogenous study population, patients undergoing bilobectomy, pneumonectomy or sublobar resection and patients without R0 resection have been excluded. Only seventeen (1.8%) patients had no standardized mediastinal lymphadenectomy according to ESTS guidelines and were kept in the analysis in order to evaluate the impact of lymphadenectomy and LN-LOR in N0 patients. Predefined data were reviewed retrospectively, focusing on resected and pathologically assessed LNs and long-term survival (Table 1). After primary data collection, all cases were reviewed by a second reviewer in order to ensure completeness and correctness of data. We assessed the following parameters: gender (dichotomous), age at resection (continuous, interval), histological subtype (categorical), tumor, node and metastases staging (ordinal), number of resected LNs (continuous, interval), number of positive LNs (continuous, interval), resected LN stations (dichotomous), positive LN stations (dichotomous), neoadjuvant treatment (dichotomous) and adjuvant treatment (dichotomous). Survival time was additionally documented (continuous, interval) and calculated from the time of resection until the end of follow-up (December 2021) or death. All patients were (re)staged using the 8th edition of the TNM staging system [2]. All patients were staged and treated in accordance with the National Comprehensive Cancer Network (NCCN) guidelines. Preoperative PET/CT and/or EBUS-TBNA were performed if clinically indicated, according to the ESTS guidelines [15]. Lobectomy and lymphadenectomy were either performed by open thoracotomy or a video-assisted approach, according to the discretion of the surgeon, and combined with video-assisted mediastinal lymphadenectomy (VAMLA) in selected cases. According to the institutional experience of center #1, VAMLA was mainly performed in left-sided tumors with enlarged 4 L LNs in order to achieve complete resection. The aim of the lymphadenectomy was to completely resect all accessible LNs en bloc without fragmenting nodes, and it was performed according to the ESTS guidelines for intraoperative lymph node staging in NSCLC [16]. The indication for surgery or any (neo)adjuvant treatment was discussed in a multidisciplinary tumor board. In case of preoperatively verified N2 LNs, patients have usually been referred to neoadjuvant treatment. Invasive mediastinal re-staging was not routinely performed. Patients with ypN0 after neoadjuvant treatment and cN3 patients have been excluded from this analysis. All variables and data of interest were extracted from the institutional patient management systems and anonymously documented in an SPSS file. Data on survival were obtained from the “Statistic Austria” institute. The study may be biased due to its retrospective design. The median follow-up was calculated to be 49 months with the reverse-censored Kaplan–Meier method and 233 events occurred during the study period.

### 2.3. Statistical Methods

LN-LOR was calculated using the formula ‘log [(positive LNs + 0.5)/(resected LNs—positive LNs + 0.5)]’ in which 0.5 was added to both the numerator and denominator to avoid singularity [17]. After calculating the respective LN-LOR for all patients, LN-LOR (continuous variable) was dichotomized by using X-tile software (version 3.6.1; Yale University, New Haven, CT, USA) based on the maximal log-rank chi-squared value, which represented the greatest group difference in survival probability. By using this cut-off value, we defined an LN-LOR low risk and high-risk group, based on its impact on OS. Categorical parameters of different patient cohorts (i.e., LN-LOR high risk and low risk) were analyzed by Chi-squared tests. Age as a continuous variable was analyzed in the different subgroups by Student’s *t*-test. Kaplan–Meier survival curves and two-sided log-rank tests were used for univariable survival analyses. The median follow-up time was estimated using the reverse-censored Kaplan–Meier method. The Cox proportional hazards model was used for univariable survival analyses to calculate the hazard ratios (HR) and corresponding 95% CI. A multivariable Cox regression model was used to evaluate the influence of LN-LOR (dichotomous) on OS and to calculate the hazard ratios (HR) and corresponding 95% CI. The multivariable model was adjusted for sex (dichotomous), age (continuous), histological subtype (categorical), pathological T factor (ordinal), pathological N factor (ordinal), overall stage (ordinal), neoadjuvant treatment (dichotomous) and adjuvant treatment (dichotomous). The *p* values are always given as two-sided and were considered statistically significant below 0.05. Metric data is always shown as median or mean and corresponding interquartile range (IQR) or, in case of OS, as median and corresponding 95% CI. Unless otherwise stated, statistical calculations were performed using SPSS Statistics version 28 (IBM Corporation, New Orchard Road, Armonk, New York 10504, USA) and GraphPad Prism version 9 (GraphPad Software, Inc. 7825 Fay Avenue, Suite 230, La Jolla, CA, USA).

All methods and results are reported according to the STrengthening the Reporting of OBservational studies in Epidemiology (STROBE) and “Statistical and data reporting guidelines for the European Journal of Cardio-Thoracic Surgery and the Interactive CardioVascular and Thoracic Surgery” guidelines [18,19].

## 3. Results

### 3.1. Patient Characteristics

Inclusion criteria resulted in a study size of 944 patients (center #1, n = 350; center #2, n = 594). In total, 529 males (56%) and 415 females (44%) with a mean age of 65 years (SD 9,1) were included. Adenocarcinoma (n = 672, 71%) was the most common histological subtype. Most patients had pT1/pT2 tumors (90%) and were node negative (76%). Accordingly, 63% of patients in this cohort were in pathological stage I. The median number of lymph nodes resected during lymphadenectomy was 12 (IQR 11), and 74 (8%) and 190 (20%) patients underwent neoadjuvant or adjuvant treatment, respectively. Basic patient characteristics are summarized in Table 1.

### 3.2. Cut-Off Analysis and Prognostic Significance of Lymph Node Log-Odds Ratio (LN-LOR)

**Figure 1:**
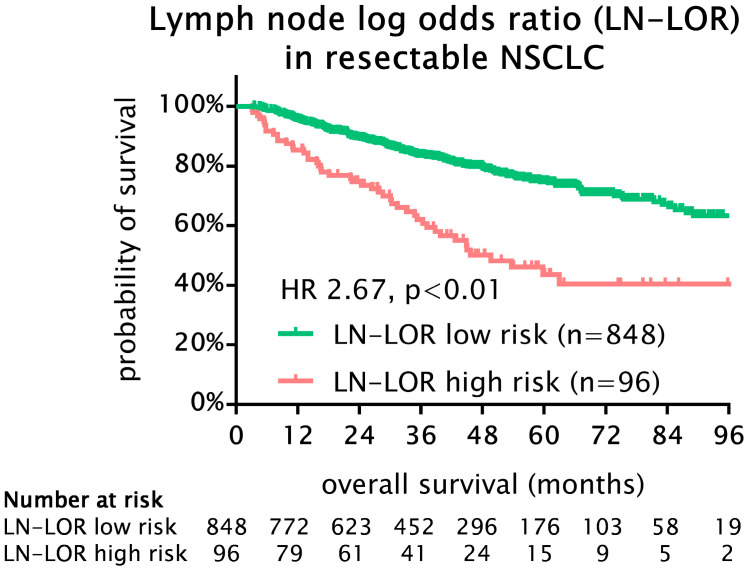
Kaplan–Meier survival analysis for OS in 944 patients with stage I-III NSCLC undergoing curative intent lobectomy. Lymph node log-odds ratio high-risk patients had a statistically significant and clinically relevant worse OS compared to low-risk patients (5-year OS 44% vs. 75%; HR 2.67, 95% CI 1.90–3.93, *p* < 0.001). NSCLC, non-small cell lung cancer; LN-LOR, lymph node log-odds ratio; HR, hazard ratio.

LN-LOR ranged from −2.03 to 1.72. X-tile software analysis identified an LN-LOR of −0.34 as the optimal cut-off value to discriminate two different prognostic groups (*p* < 0.001, chi-squared 41.26). According to OS, we defined an LN-LOR low risk (n = 848; 90%) and an LN-LOR high risk (n = 96; 10%) group. Notably, LN-LOR high-risk patients had a statistically significant and clinically relevant worse OS compared to low-risk patients (5-year OS 44% vs. 75%; HR 2.67, 95% CI 1.90–3.93, *p* < 0.001) (Figure 1).

### 3.3. Association of LN-LOR with Clinical and Pathological Factors

We did not observe a statistically significant correlation between LN-LOR and clinical factors such as age or sex. Furthermore, LN-LOR did not correlate with pathological factors such as histological subtype or pT stage. As to be expected, patients in higher pN (*p* = 0.001) and overall stages (*p* = 0.001) tended to be in the LN-LOR high-risk group, respectively. Notably, when we correlated LN-LOR risk groups with different affected anatomical LN locations, we found that patients with positive station 7 LNs tended to be more often in the LN-LOR high-risk group (18% vs. 2%, *p* = 0.048) (Table 2). We did not observe this tendency among other affected lymph node stations.

### 3.4. Survival Analysis

**Figure 2:**
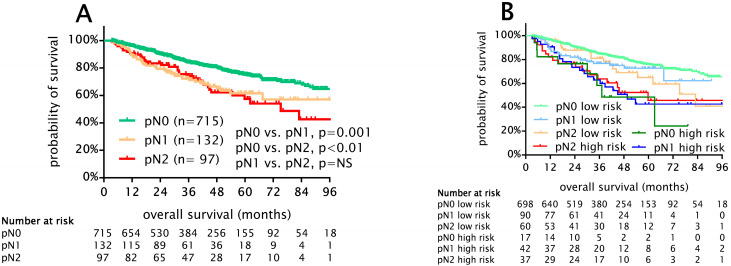
Kaplan–Meier survival analysis for OS in 944 patients with stage I-III NSCLC undergoing curative intent lobectomy. (**A**) Survival analysis according to pN subgroups. Importantly, pN0 patients exhibited significantly improved OS compared to pN1 (*p* = 0.001) and pN2 (*p* < 0.01) and, moreover, OS of pN1 and pN2 patients was almost similar (5-year OS 61% and 58%, *p* = 0.79). (**B**) Survival analysis according to pN subgroups stratified by LN-LOR. LN-LOR, lymph node log-odds ratio; NS, not significant.

**Figure 3:**
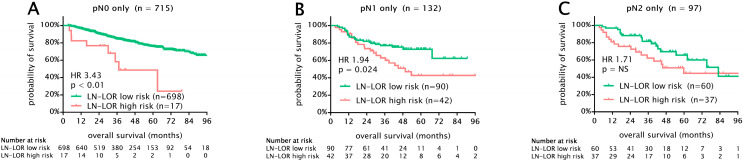
Kaplan–Meier survival analysis for OS in 944 patients with stage I-III NSCLC undergoing curative intent lobectomy. Survival analysis stratified by pN categories and LN-LOR risk groups. (**A**) OS in pN0 patients only. LN-LOR high risk was associated with significantly worse OS (5-year OS 76% vs. 48%, *p* < 0.01) among pN0 patients. Importantly, 17 patients in the LN-LOR high-risk group did not undergo adequate lymphadenectomy. (**B**) OS in pN1 patients only. LN-LOR high risk was associated with significantly worse OS (5-year OS 73% vs. 41%, *p* = 0.024) among pN1 patients. (**C**) OS in pN2 patients only. We found a non-significant tendency for worse OS in LN-LOR high-risk patients in the pN2 subgroup (5-year OS 67% vs. 45%, *p* = 0.11). Importantly, pN1 and pN2 LN-LOR low risk (median OS 87 and 83 months) and pN1 and pN2 LN-LOR high-risk patients (median OS 50 and 59 months) had similar survival. LN-LOR, lymph node log-odds ratio; NS, not significant; HR, hazard ratio.

The median overall survival of the whole cohort was 90 months (95% CI 87–94). The results of a univariable survival analysis of standard prognostic factors are given in Table 3. When survival was analyzed with regard to different pN subgroups, we found that patients with pN0 had superior OS compared to those with pN1 (HR 1.8, 95% CI 1.29–2.52, *p* = 0.01) and pN2 disease (HR 1.89, 95% CI 1.31–2.73, *p* = 0.01). Interestingly, in this cohort, the survival of pN1 and pN2 patients was similar (5-year OS 61% and 58%, *p* = 0.79) (Figure 2A). Next, we combined the anatomical pN descriptor with LN-LOR risk groups and found that LN-LOR high-risk patients exhibit clinically relevant worse OS among all pN0-2 subgroups (5-year OS low risk vs. high risk, pN0 (76% vs. 48%, *p* < 0.01), pN1 (73% vs. 41%, *p* = 0.024), pN2 (67% vs. 45%, *p* = 0.11)) (Figure 3A–C). Importantly, pN1 and pN2 LN-LOR low risk (median OS not reached and 83 months) and pN1 and pN2 LN-LOR high-risk patients (median OS 50 and 59 months) had similar overall survival (Figure 2B).

In univariable survival analysis we found that, besides LN-LOR, clinicopathological parameters such as sex, age, histological subtype, pT stage, pN stage, overall stage and the use of adjuvant treatment were significant factors for OS (Table 3). We further performed a multivariable Cox regression analysis adjusted for parameters such as sex, age, histological subtype, pT stage, pN stage, overall stage, use of neoadjuvant treatment and use of adjuvant treatment. Importantly, multivariable analysis revealed that LN-LOR remained as an independently significant factor for OS (low-risk group, HR 0.48, 95% CI 0.32–0.72, *p* = 0.001) (Table 3).

## 4. Discussion

The traditional TNM staging system is a well established and reliable tool to guide treatment and estimate prognosis in NSCLC [1,2,20,21]. However, the current pN staging descriptors are based on anatomical LN locations only and do not take the number of resected or the number of positive LNs into account. Notably, it has been previously shown that the number of resected LNs, the number of positive LNs, the ratio of positive/resected LNs (LN ratio) and the number of positive LN stations may significantly influence OS, and thus considering only anatomical LN locations might unreliably predict outcomes in resectable NSCLC [1,5,22,23,24,25]. In addition, the LN ratio has been shown to be a promising factor in order to accurately estimate prognosis in resectable NSCLC with pN1 and pN2 disease [6,7,25,26]. However, resection of fewer LNs negatively impacts the value of the LN ratio, and moreover, there is no value in pN0 patients. Unlike the LN ratio, the log-odds ratio of positive and resected lymph nodes is a well described measure that encompasses the numbers of resected, negative and positive LNs, and it therefore has advantages over other node-based descriptors and the current N staging system for lung cancer [27,28]. In the present retrospective cohort study, we investigated the prognostic value of LN-LOR regarding different pN subgroups in 944 patients undergoing curative intent lobectomy for stage I-III NSCLC. Notably, we found that LN-LOR was superior to the current anatomical N descriptor in adequately stratifying patients into different and distinct prognostic groups. In order to allocate patients into two groups according to OS probability, X-tile software analysis was used to identify an adequate cut-off value for LN-LOR. In our study, an LN-LOR of −0.34 was found to be the optimal cut-off for OS, being applicable to all pN0–2 subgroups. Previous studies on LN-LOR in NSCLC have reported different cut-off values. In two studies, there was a single cut-off value: −1.142 and 0.26 [28,29]. In one study, there were four different groups based on values ranging from −2.10 to 1.74 [27]. Seven prognostic groups were identified in another recent study, where values ranged from −6 to 2 [30]. Of note, different inclusion criteria were applied among these studies (i.e., exclusion of pN0 patients) and further research is warranted to define a commonly accepted cut-off in order to include LN-LOR to future NSCLC staging systems.

LN-LOR might be superior to the current N descriptors in reflecting the lymphatic disease burden and its influence on OS. As expected, we found that patients with higher pN stages and higher overall stages tended to be in the LN-LOR high-risk group. However, we also found that LN-LOR was additionally significantly associated with the presence of affected station 7 LNs. This finding might be partly explained by the mediastinal lymphatic vessel anatomy, where station 7 LNs could play a central role in mediastinal lymphatic cancer spreading and thus could be associated with a higher total number of affected LNs. To the best of our knowledge, this is the first study observing this possible association. Moreover, a recent study investigating 4797 patients with stage IIIA-N2 resected NSCLC from the Surveillance, Epidemiology and End Results (SEER) database has additionally shown that LN-LOR not only exhibited the best prognostic performance in predicting OS, but also could identify patients who benefit from PORT [31].

Of note, we found that the survival of pN1 and pN2 patients was almost similar in our cohort of radically treated early-stage NSCLC. This finding is inconsistent with a large number of previous studies showing that pN2 is significantly associated with inferior prognosis compared to pN1. In contrary, however, other studies have clearly demonstrated that a subset of radically treated pN2 patients can have improved OS, with survival rates similar to stage II patients [1,32]. Furthermore, we could show in the present study that using LN-LOR was superior in defining different prognostic groups in all pN subsets, and moreover, that prognosis in patients with pN1 and pN2 disease can be further stratified by accurately evaluating the LN disease burden using LN-LOR.

In the current TNM-8 staging system, it is suggested that at least six LNs from at least three different stations should be resected for adequate staging. However, according to various studies this is not an appropriate number [1,33]. Among most studies, ten was the most frequent minimal value of resected LNs that was considered prognostically significant [33]. More extensive LN resection might be more beneficial, as it provides a better information on the actual disease burden, leading to a more accurate staging and eventually possibly improved survival rates. To the best of our knowledge, the number of resected LNs impacts the prognosis also in the subgroup of pN0 patients. In our study we found that among N0, those without an adequate lymphadenectomy and consequently being allocated to the LN-LOR high-risk group had a significantly worse OS. This finding might be explained by a possibly unrecognized hilar or mediastinal LN involvement due to improper lymphadenectomy. Accordingly, the findings of our study additionally highlight the importance of a proper lymphadenectomy also in patients without clinical nodal involvement. Moreover, this study supports the value of extensive LN dissection. The issue of whether extensive LN dissection itself has any therapeutic effect is still highly debated, and to the best of our knowledge, there is still insufficient scientific evidence to support this assumption. Considering the physiological lymphatic drainage particularly of the lower lobes to the contralateral mediastinum and the limited surgical access to groups 2 L and 4 L via the left pleural cavity, VAMLA was mainly performed along with left-sided lobectomies in our cohort.

Our study has several limitations, partly due to its retrospective nature. Patients with different histological subtypes of NSCLC have been included, which could possibly bias our results. Furthermore, we have not taken the possible influence of tumor mutations by means of molecular pathology into account. In addition, information on recurrence was not available and recurrence-free survival could not be investigated. Lastly, the categorization of continuous variables, as performed in our study, bears the risk of loss of information and may be debated.

## 5. Conclusions

In summary, this retrospective two-center analysis shows that LN-LOR is significantly associated with OS and might better reflect the biological behavior of the disease. According to our results, the anatomical location of involved LNs (i.e., N descriptor) might be inferior in estimating prognosis compared to the number of positive LNs. This finding may additionally support the value of extensive LN dissection.

## Figures and Tables

**Table 1:** Basic clinical and pathological parameters of 944 patients undergoing curative intent lobectomy for stage I-III NSCLC.

Parameter		n	%
Sex	female	415	44%
	male	529	56%
Age	<65	404	43%
	≥65	540	57%
Histological subtype	Adenocarcinoma	672	71%
	Squamous cell carcinoma	211	22%
	Other	61	7%
Pathologic T Stage	pT1	487	52%
	pT2	355	38%
	pT3	81	9%
	pT4	21	2%
Pathologic N Stage	pN0	715	76%
	pN1	132	14%
	pN2	97	10%
Overall Stage	I	599	63%
	II	212	22%
	III	133	14%
Total number of resected lymph nodes	median (IQR)	12 (11)	
Neoadjuvant treatment	no	870	92%
	yes	74	8%
Adjuvant treatment	no	754	80%
	yes	190	20%

n, number of patients; IQR, interquartile range.

**Table 2:** Association of lymph node log-odds ratio (LN-LOR) risk groups with clinical and pathological parameters in a cohort of 944 patients undergoing lobectomy for stage I-III NSCLC.

		All Patients	LN-LOR Low Risk		LN-LOR High Risk		
		n	n	%	n	%	*p*
Sex	female	415	368	43%	47	49%	0.25
	male	529	480	57%	49	51%	
Age	<65	404	363	43%	41	43%	0.138
	≥65	540	485	57%	55	57%	
Histological subtype	ADC	672	597	70%	75	78%	0.167
	SQC	211	196	23%	15	16%	
	Other	61	55	7%	6	6%	
pT Stage	pT1	487	442	52%	45	47%	0.36
	pT2	355	316	37%	39	41%	
	pT3	81	73	9%	8	8%	
	pT4	21	17	2%	4	4%	
pN Stage	pN0	715	698	82%	17	18%	**0.001**
	pN1	132	90	11%	42	44%	
	pN2	97	60	7%	37	39%	
Stage	I	599	585	69%	14	15%	**0.001**
	II	212	173	20%	39	41%	
	III	133	90	11%	43	45%	
Station 7 positive	no	870	830	98%	79	82%	**0.048**
	yes	74	18	2%	17	18%	

Statistically significant *p* values are highlighted in bold letters. LN-LOR, lymph node log-odds ratio; n, number of patients; ADC, adenocarcinoma; SQC, squamous cell carcinoma.

**Table 3:** Univariable and multivariable Cox regression survival analysis for clinical and pathological factors in a cohort of 944 patients undergoing lobectomy for stage I-III NSCLC.

	Univariable Analysis				Multivariable Analysis			
	HR	95% CI		*p*	HR	95% CI		*p*
		Lower	Upper			Lower	Upper	
Sex (male)	0.60	0.49	0.79	**0.001**	1.65	1.25	2.19	**0.001**
Age (continuous)	1.01	1.00	1.03	**0.023**	1.02	1.00	1.03	**0.020**
Histological subtype				**0.004**				**0.003**
ADC	-	-	-		-	-	-	
SQC	1.35	1.01	1.82	**0.046**	1.23	0.91	1.67	0.176
Other	1.94	1.27	2.96	**0.002**	2.11	1.35	3.28	**0.001**
pT Stage				**0.001**				0.25
pT1	-	-	-		-	-	-	
pT2	1.52	1.15	2.01	**0.003**	1.33	0.99	1.78	0.060
pT3	2.21	1.45	3.37	**0.000**	1.48	0.85	2.55	0.164
pT4	2.27	1.14	4.49	**0.019**	1.64	0.62	4.34	0.31
pN Stage				**0.001**				0.68
pN0	-	-	-		-	-	-	
pN1	1.80	1.29	2.52	**0.010**	0.83	0.49	1.39	0.47
pN2	1.89	1.31	2.73	**0.010**	1.01	0.40	2.55	0.99
Stage				**0.001**				**0.013**
Stage I	-	-	-		-	-	-	
Stage II	2.27	1.69	3.04	**0.001**	1.91	1.23	2.98	**0.004**
Stage III	2.19	1.56	3.08	**0.001**	1.49	0.60	3.73	0.39
Neoadjuvant treatment (yes)	1.37	0.90	2.07	0.140	1.33	0.86	2.07	0.199
Adjuvant treatment (yes)	1.39	1.04	1.87	**0.029**	0.98	0.70	1.37	0.89
LN-LOR low	0.38	0.28	0.53	**0.001**	0.48	0.32	0.72	**0.001**

Statistically significant *p* values are highlighted in bold letters. HR, hazard ratio; CI, confidence interval; ADC adenocarcinoma; SQC, squamous cell carcinoma; LN-LOR, lymph node log-odds ratio.

## Data Availability

Data are available on reasonable request from the authors.

## Abbreviations

ADC	adenocarcinoma
CI	confidence interval
cN	clinical lymph node involvement
ESTS	European society of Thoracic Surgeons
HR	hazard ratio
IQR	interquartile range
LN	lymph node
LN-LOR	lymph node log-odds ratio
LNR	lymph node ratio
NSCLC	non small cell lung cancer
OS	overall survival
pN	pathological lymph node involvement
PORT	post operative radiotherapy
SD	standard deviation
STROBE	STrengthening the Reporting of OBservational studies in Epidemiology
SQC	squamous cell carcinoma
TNM	tumor node metastasis
VAMLA	video-assisted mediastinal lymphadenectomy
